# Unrealistic Optimism: East and West?

**DOI:** 10.3389/fpsyg.2013.00006

**Published:** 2013-02-13

**Authors:** Mary Sissons Joshi, Wakefield Carter

**Affiliations:** ^1^Department of Psychology, Social Work and Public Health, Oxford Brookes UniversityOxford, UK

**Keywords:** unrealistic optimism, culture, India, England, self-enhancement

## Abstract

Following Weinstein’s (1980) pioneering work many studies established that people have an optimistic bias concerning future life events. At first, the bulk of research was conducted using populations in North America and Northern Europe, the optimistic bias was thought of as universal, and little attention was paid to cultural context. However, construing unrealistic optimism as a form of self-enhancement, some researchers noted that it was far less common in East Asian cultures. The current study extends enquiry to a different non-Western culture. Two hundred and eighty seven middle aged and middle income participants (200 in India, 87 in England) rated 11 positive and 11 negative events in terms of the chances of each event occurring in “their own life,” and the chances of each event occurring in the lives of “people like them.” Comparative optimism was shown for bad events, with Indian participants showing higher levels of optimism than English participants. The position regarding comparative optimism for good events was more complex. In India those of higher socioeconomic status (SES) were optimistic, while those of lower SES were on average pessimistic. Overall, English participants showed neither optimism nor pessimism for good events. The results, whose clinical relevance is discussed, suggest that the expression of unrealistic optimism is shaped by an interplay of culture and socioeconomic circumstance.

## Introduction

Ever since its original demonstration by Weinstein (1980) a great deal of empirical work and theoretical attention has been devoted to the phenomenon of unrealistic optimism. The term refers to a bias whereby “people rate negative events as less likely to happen to themselves than to the average person and positive events as more likely to happen to themselves than to the average person” (Harris and Hahn, 2011, p. 135). In accounting for the optimistic bias, many researchers have seen it as an instance of the more general “self-enhancing bias.” Further, far from being maladaptive an optimistic bias even if “unrealistic” has long been held to be associated with psychological well-being (Taylor and Brown, 1988), and conversely, a pessimistic bias is thought to be involved in depression (Abramson et al., 1978; Miranda and Mennin, 2007).

### Self-enhancement, unrealistic optimism, and culture

The majority of the early research on unrealistic optimism in the 1980s and 1990s was conducted on participants in the USA (q.v. meta-analysis of a perceived control and optimistic bias, Klein and Helweg-Larsen, 2002) and it was some time before psychologists questioned the ubiquity of the unrealistic optimism bias. Noting that the self-enhancing bias thought to underlie unrealistic optimism is itself not universal (Markus and Kitayama, 1991) and considerably less prevalent in cultures with an interdependent construal of self, Heine and Lehman (1995) investigated unrealistic optimism in Canada and Japan. Canadian respondents showed optimism on both positive and negative items whether they were making relative or absolute judgments about their likelihood of experiencing these events in comparison to their peers. In contrast, Japanese respondents only showed unrealistic optimism in one specific domain, i.e., relative likelihood estimated for negative events and Heine and Lehman (1995) speculated that this “specific pocket” of optimism for their Japanese respondents was related to a possible methodological artifact in their study. Heine and Lehman (1995) concluded overall that “self-enhancing biases (such as unrealistic optimism) are, for the most part, absent from the Japanese motivational repertoire because the consequent attention to the individual that self-enhancement engenders is not valued in interdependent cultures” (p. 595). However, some studies have found evidence of optimistic bias in non-Western interdependent cultures. For example, Chang et al. (2001) found an optimistic bias regarding the likelihood of negative everyday events not only in the responses of European Americans but also of Japanese. Although Chang et al. (2001) found no evidence of an optimistic bias for positive events among European Americans, they did find evidence of a pessimistic bias for positive events among Japanese. In a subsequent study Chang and Asakawa (2003) asked respondents to compare their likelihood of experiencing atypical events in comparison not to a “similar other” as is usual in comparative optimism studies, but to a sibling. With this alteration of focus, Chang and Asakawa (2003) now found European Americans displaying an optimistic bias regarding both positive and negative events, and the Japanese showing no bias in either direction for positive events and a pessimistic bias for negative events.

### Methodological issues: Measurement and sampling

More recently, work has differentiated between cognitive biases (such as informational egocentrism) and motivational biases (such as self-enhancement), and suggested that the emergence of these biases depends on the method used to examine risk perception (Chambers and Windschitl, 2004; Harris and Hahn, 2011). Asking participants to assess their own likelihood of experiencing an event, and assess separately the likelihood of a specific comparison group experiencing the same event, is known as the indirect method. Asking participants a single question where they must compare their likelihood of experiencing a particular event in comparison to another group/type of person (e.g., for a student sample – “how likely are you to have a heart attack before the age of 40, compared to the average student of your age/sex”) is known as the direct method and is thought to exacerbate respondents’ susceptibility to cognitive biases, such as statistical regression to the mean and subjective assessment of risk frequency (Lichtenstein et al., 1978). In a study where participants made direct comparison likelihood estimates, both Japanese and USA respondents showed unrealistic optimism about infrequent negative events and unrealistic pessimism about frequent negative events (Rose et al., 2008). Proposing that base rate biases are more detrimental to the judgment of the probability of others experiencing events than they are to probability judgments concerning the self, the authors interpret the optimistic bias shown in their study as being related more to a “culture-free” cognitive bias than to a “culture-specific” motivational bias. In the same study cultural differences did emerge when the participants made indirect judgments and under these conditions Japanese respondents showed considerably less optimism than USA respondents.

In summary, to date research does suggest that the tendency to self-enhance and show unrealistic optimism is very robust among Westerners, and not subject to methodological nuance. In contrast, non-Western samples show far less self-enhancement and such unrealistic optimism as is shown is limited to certain methods (Heine and Hamamura, 2007). However, a striking aspect of the unrealistic optimism literature is that the cultures used in the studies are somewhat restricted. The most frequently used exemplars of Western cultures are USA and Canada, and the most frequently used exemplars of non-Western cultures are East Asian cultures (such as Japan and Korea; Heine and Hamamura, 2007; Klein and Helweg-Larsen, 2002). East Asian cultures are paradigmatic examples of cultures with an interdependent rather than an independent construal of self, but they may also have related characteristics which play a part in their lack optimistic bias. For example, it has been noted that the weakness or absence of self-enhancing motivations among people of East Asian descent is found “specifically (among) those that participate in Confucian cultures” (Heine and Hamamura, 2007, p. 5). Research is needed to investigate whether members of other cultures with an interdependent concept of self also show a lack of optimistic bias when judging their relative risk of experiencing negative and positive events.

### Unrealistic optimism and India

India is the location for a set of cultures characterized as having an interdependent concept of self (Roland, 1988; Mascalo and Bhatia, 2002; Mascalo et al., 2004)^1^. Many aspects of parent-child interaction in India are frequently used to exemplify the development of the interdependent self (Shweder et al., 1995; Saraswathi and Ganapathy, 2002; Kapadia and Miller, 2005). Further, two of the classic studies which illustrate the contrasting attributional styles of those who have independent and interdependent concepts of self drew their non-Western samples from India (Shweder and Bourne, 1982; Miller, 1984). Closely related to the distinction between independent and interdependent concepts of self is the description of cultures as individualistic or collectivist, and just as East Asian cultures have been categorized as collectivist, so too has India (Hoftsede, 1980; Triandis and Suh, 2002)^2^. While it has been argued that “the Indian psyche” is best understood as individualistic as well as collectivistic (Sinha and Tripathi, 1994; Kumar, 2004), it can confidently be said that, in many aspects of their philosophical position and emphasis on social harmony and hierarchy, cultures in India (spanning a variety of religions including Hinduism and Islam) share much with East Asian Confucian cultures (Laungani, 2007)^3^. Therefore, like East Asians, Indian participants might well be expected not to show unrealistic optimism.

As explicated above, discussion of cultural differences in unrealistic optimism has focused on contrasting North Americans and East Asians, and in particular has debated the motivating force of self-enhancement. But self-enhancement is only one of the motivational mechanisms thought to be involved in unrealistic optimism. Another mechanism is the illusion of control (Langer, 1975). While self-enhancement and the illusion of control can both be regarded as instances of a more general self-serving bias, particularly due to their co-occurrence in the motivational repertoire of Westerners, it is useful to conceptualize these variables as separate. A study by McKenna (1993) indicated that, among UK participants, unrealistic optimism was not so much a generalized expectancy for a positive outcome but more closely related to the extent to which the participant had control over the outcome. If the illusion of control is heavily involved in unrealistic optimism, one would expect to find less unrealistic optimism among respondents whose culture places less value on control. Among cultures in India, Hinduism in particular has been portrayed as such a culture. From a psychoanalytic perspective, Kakar writes “With the Hindu emphasis on man’s inner limits, there is not that sense of urgency and struggle against the outside world, with the prospects of sudden metamorphoses and great achievements just around the corner, that often seems to propel Western lives” (Kakar, 1978, p. 49). Laungani suggests that “The law of karma allows a Hindu to accept with passivity and fortitude the vicissitudes of life” (Laungani, 2007, p. 200). Empirical studies of response to misfortune, such as chronic illness, suggest that the connection between positive adjustment, perceived control and lay causal reasoning so often found in the West (Taylor and Brown, 1988) is not found in Indian patients (q.v. Joshi, 1995). A series of studies by Savani and colleagues also indicate that the discourses of control and “choosing according to one’s personal preferences may not be as important to the experience of agency for Indians as it is for North Americans” (Savani et al., 2008, p. 861; Savani et al., 2010, 2011).

### Unrealistic optimism: Theories and applications

The discussion of culture, unrealistic optimism, and the self-enhancement bias has predominantly been concerned with notions of the independent/interdependent self in individualistic/collectivist societies. An entirely different explanation for self-enhancement has recently been suggested by Loughnan et al. (2011). In their 15 nation study, differences between nations in the extent of self-enhancement (evidenced by people’s tendency to see themselves as better than the average person on a variety of personality traits and values) were better predicted by the extent of income inequality in those countries than by individualism/collectivism scores. For example, in Loughnan et al.’s (2011) data set, Korea and Peru have very different profiles despite both being at the collectivist end of the I-C scale (Hoftsede, 2001). Korea has low income inequality (as measured by the Gini coefficient) and low self-enhancement scores; Peru has high income inequality and high self-enhancement scores (the latter being far higher than those shown by the USA in the same data set). In explaining the apparent connection between self-enhancement and income inequality, Loughnan et al. (2011) suggest that self-enhancement relates to factors that result from socioeconomic differences, such as the drive to compete for social superiority thought by Wilkinson and Pickett (2010) to be characteristic of living in a socially and economically unequal society.

A major limitation of much research in comparative optimism, and indeed in social psychology in general, is its reliance on student participants (Henrich et al., 2010). Few if any studies have compared rates of unrealistic optimism and self-enhancement for different socioeconomic groups within a culture. Loughnan et al. (2011) do raise the issue of whether self-enhancement might vary not only between societies but within societies. Their own sample was drawn predominantly from students who they suggest “might often find themselves in situations in which their social standing in actually better than the average person’s, an effect which would be more pronounced in societies with more income inequality” (Loughnan et al., 2011, p. 1257). As Loughnan et al. (2011) point out, comparative optimism studies do attempt to “guard against this confound” (p. 1257) by having participants compare themselves with the average other from their own group. Given the current study’s interest in the relationship between optimism and inequality, it will be important to sample from more than one socioeconomic group.

In addition to addressing theoretical issues concerning culture and unrealistic optimism, the results of the study may have some bearing on applied health issues. Unrealistic optimism and pessimism are event-specific biases “manifested by individuals, but measured at the level of the group” (Jansen et al., 2011, p. 2). In the case of epidemiologically common risks, high levels of optimism are of clinical interest as they may well discourage disease preventive action in those at risk (Sweeny et al., 2006; Schacter and Addis, 2007). Cardiovascular disease (and coronary heart disease in particular) is the main cause of death in England (British Heart Foundation, 2012). Recently, heart disease has also been established as the foremost cause of death in India (Jha and Laxminarayan, 2009). Its even higher prevalence and pattern of earlier onset in India than in industrialized nations has been attributed to the interplay of genetic and lifestyle factors such as fatty diet, smoking, and lack of exercise (Kaul and Bhatia, 2010). High levels of unrealistic optimism in England or India in respect of items such as “risk of a heart attack” would be concerning as optimism is likely to stand in the way of people making health promoting behavioral changes.

Much less is known about unrealistic pessimism than about unrealistic optimism. Such work as there is has focused on unrealistic pessimism about negative events, such as serious illness, and attempted to relate pessimism to low uptake of screening or lack of engagement with treatment (Lerman and Schwartz, 1993; Klein et al., 2010). Unrealistic pessimism, particularly about the likelihood of experiencing positive events, may be indicative of depression (Miranda and Mennin, 2007. Further, if rates of unrealistic pessimism across a number of different types of event are high, this may serve as useful indirect measure of depression at the population level, especially in social cultural contexts where mental illness may be stigmatized, and depression is often presented in somatic as opposed to psychological terms (Bhugra and Mastrogianni, 2004; Jadhav et al., 2007; Ryder et al., 2008).

The current study sets out to explore unrealistic optimism in India and in England among two socioeconomic groups. There are two closely related grounds for thinking that Indian participants will show less unrealistic optimism than English participants. Following Heine and Hamamura (2007) and taking unrealistic optimism to be an example of self-enhancement it can be expected that Indian participants (likely to have an interdependent concept of self typical of Asian cultures) will show less unrealistic optimism than English participants. Following McKenna (1993) and taking unrealistic optimism to be an example of the illusion of control, it can also be expected that Indian participants (likely to place less importance on control) will show less unrealistic optimism that English participants. In contrast, in the light of Loughnan et al.’s (2011) demonstration that income inequality relates to self-enhancement, then given that India and UK have very similar Gini coefficient income inequality scores, there are grounds for expecting that Indian and English participants will show very similar rates of unrealistic optimism^4^. Given the opposing arguments which characterize the background literature, no directional predictions will be made.

## Materials and Methods

### Instrument

In the spirit of a derived etic approach (Berry, 1989), Weinstein’s (1980) future event list, developed for use in his original study using female students at Rutgers University, USA, was taken as the starting point for the current study. Giving “being injured while skiing” as an example, Weinstein cogently pointed out that even in one culture there can be “no obvious relevant precondition that would make any event relevant to only a limited number of people” (1980, p. 809). Pilot work was conducted to develop a list of good and bad events suitable for use in India and England. It was immediately apparent that some of Weinstein’s original items would not be culturally appropriate in England or India. For example “Car turns out to be a lemon” (Weinstein, 1980, p 810) is not only expressed in the idiom of American slang but is unsuited to a population where car ownership is considerably less common than in the USA.

It was considered important in the development of the list of events to be used in the current study to give priority to the Indian rather than the English future participants, on the assumption that Weinstein’s items would be even less appropriate to the former rather than the latter cultural group. Three male and three female English-Marathi bilingual Indian informants were recruited in Mumbai to evaluate the Weinstein future event list (shortened to 22 items to exclude relatively trivial events such as “having gum problems” and “in bed ill for two or more days” and exclude items only relevant to students such as “graduating in top third of class”). The informants rated 12 items as suitable (or requiring only minimal rewording) and suggested 10 new items to capture good and bad events they regarded as missing from the original list (See Tables 2– 5 for the list of events used).

Mixing positive and negative items, the 22 events were placed in a random order. Participants were asked to assess on a scale from 0, 10, 20, …, 100%) the chance of each event happening in their own life, and in the lives of people like them. There were four versions of the questionnaire, varying order of self-other rating and varying whether the example given was optimistic or pessimistic. In versions 1 and 2, for each item, the participant firstly rated his/her own chance and then rated the chances for people like themselves. In versions 3 and 4, for each item, the participant firstly rated the chances for people like themselves, and then rated his/her own chance. In versions 1 and 3, the instruction on the questionnaire read “The chances of an event occurring in your life may be the same or different from the chances of the event occurring in the lives of people like you. For example, you may think that the chance of your having a heart attack is 30% and the chance of people like you having a heart attack is also 30%; or you may think that because your health is very good the chance of your having a heart attack is only 10% and the chance of people like you having a heart attack is 30%.” In versions 2 and 4, the instruction read “The chances of an event occurring in your life may be the same or different from the chances of the event occurring in the lives of people like you. For example, you may think that the chance of your having a heart attack is 30% and the chance of people like you having a heart attack is also 30%; or you may think that because your health is not good the chance of your having a heart attack is 70% and the chance of people like you having a heart attack is 30%.”

The questionnaire was translated into Marathi by one fluent Marathi/English speaker, and back translated into English by another fluent Marathi/English speaker. In the case of both speakers, Marathi was their mother tongue. Following the “committee assessment procedure” (Harkness and Schoua-Glusberg, 1998) apparent problems were resolved by a panel of three additional Marathi/English speakers, comprising one social scientist and two professional survey designers.

### Participants

In England, 200 administrative staff at Oxford Brookes University were invited by letter to respond to the questionnaire. They had been randomly selected from the complete staff list of administrative employes falling within the age range 25–55 years, who numbered 414. Replies were received from 87 people (29 males, 58 females) constituting a 44% response rate among those asked (and 21% of the relevant work-force). The sample was divided into two socioeconomic status (SES) groups contrasted in terms of education and occupation. Forty participants (17 males, 23 females) had been educated to first degree level or beyond and were in lower managerial/professional occupations. Forty-seven participants (12 males, 35 females) did not have degree level qualifications and were in “intermediate”/secretarial/clerical occupations (Office for National Statistics, 2010).

In India, participants were recruited by face-to-face invitation and specific residential locations were targeted in an attempt to match the type of occupations sampled in England. To recruit participants in lower managerial/professional occupations, a local researcher went door-to-door in several housing associations in Kothrud, an upper-middle income area of Pune, India. Where a married couple participated the researcher ensured that the questionnaires were filled in separately. Participants typically reported that they had higher degree qualifications and were employed in the professions as engineers, accountants, and teachers. To recruit participants in clerical occupations, a local researcher went door-to-door in several housing associations in a lower middle income area of Kurla, Greater Mumbai. Participants typically reported that they had no education beyond senior school leaving age (12th standard) and were employed in clerical occupations in government or private sector organizations. One hundred participants (50 males, 50 females) were recruited in Pune, and 100 respondents (54 males, 46 females) were recruited in Mumbai. The local researchers reported that no one approached declined to take part as “they were intrigued” by the questionnaire.

Across the entire sample, the participants’ average age was 38.9 years (*SD* 9.7) and this did not vary by SES or nationality. Using standard UK Census categories, all the participants in England classified themselves as of “white” ethnicity, and as Christian or of “no religion”^5^. The local researchers reported that all the participants in Pune and Mumbai were Indian nationals, Hindu, and had Marathi as their mother tongue.

### Event evaluation

A second set of 92 participants was recruited to evaluate the 11 good and 11 bad events in terms of their controllability and their desirability. Thirty-two (16 males, 16 females) of these participants were employees at Oxford Brookes University, England: 16 in managerial and 16 in clerical posts. Sixty participants were recruited in India: 30 each (15 male, 15 female) from the two different socioeconomic areas used in the main study. For judgments about controllability participants were asked to rate each good item using a 4-point scale – It is impossible/slightly possible/reasonably possible/certainly possible to make this happen, and rate each bad event using a 4-point scale – It is impossible/slightly possible/reasonably possible/certainly possible to prevent this happening. Participants were asked to rank the 11 good events from the very best to the least good event, and the 11 bad items from the very worst event to the least bad event. The order of these four tasks was varied between participants.

## Results

### Summary of key analyses

In order to compare the overall rates of unrealistic optimism between India and England among two socioeconomic groups, two 2 × 2 (nationality by SES) analyses of variance were conducted – one for comparative risk perceptions averaged across bad events, and one for comparative risk perceptions averaged across good events. To investigate whether rates of unrealistic optimism varied by specific event two kinds of analyses were conducted. For each event one sample *t* tests were used to establish whether each group’s comparative risk estimate differed from zero (where zero would indicate no difference in expectation of an event happening to self in comparison to the event happening to a similar other). In addition, χ^2^ tests were used to compare the ratio of optimists to pessimists within each group to a chance distribution. Finally, based on the results of a factor analysis of event frequency, controllability and desirability, their possible confounding role was investigated using a series of multiple regressions to assess the relationship of these factors to comparative optimism for bad and good events for each of the four participant groups.

### Optimism/pessimism averaging across types of item

**Figure 1:**
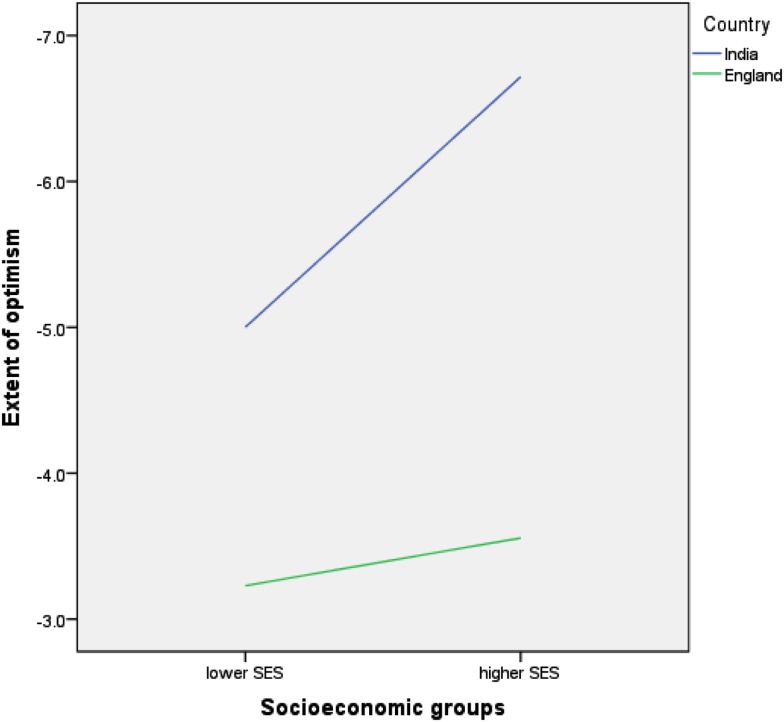
**Relative optimism for bad events**.

Table 1 displays participants’ comparative estimates of the chances of good and bad events happening in their own lives in comparison to the chances of those events happening in the lives of people like themselves. Two 2 × 2 (nationality by SES) analyses of variance were conducted, i.e., good and bad events were analyzed separately. Comparative optimism was shown for bad events, with Indian participants showing higher levels of optimism than English participants [*F*(1,283) = 5.62, *p* = 0.018]. Averaging across the 11 negative items, Indians assessed their chances of experiencing bad events as 5.9% less than people like them. This number emanates from 77% assessing their chances across events as being on average 8.4% less than others, 10% estimating their chances to be the same as others, and 13% estimating their chances to be 4.5% worse than others. English participants on average assessed their chances of experiencing bad events as only 3.4% less than others. This number emanates from 71% assessing their chances across events as being on average 6.9% less than others, 7% estimating their chances to be the same as others, and 22% estimating their chances to be 7% worse than others. There was no effect for SES [*F*(1,283) = 0.96, *p* = 0.327] nor was there an interaction between SES and nationality [*F*(1,283) = 0.45, *p* = 0.505]. See Figure 1. Optimism scores for bad events did not vary by gender or by task order/questionnaire version.

**Table 1:** **Participants’ average estimates of the chances of events happening in their own lives in comparison to the chances of those events happening in the lives of people like themselves**.

Events			Bad+		Good++	
	Socioeconomic status	N	M	SD	M	SD
India	Lower	100	−5.00***	8.23	−2.60**	11.55
	Higher	100	−6.72***	8.32	3.66**	10.61
	All	200	−5.86***	8.30	0.53	11.50
England	Lower	47	−3.23**	7.97	1.62	9.77
	Higher	40	−3.56**	7.16	−1.25	10.59
	All	87	−3.38***	7.56	0.46	11.10

*^+^On bad events, a negative score indicates comparative optimism*.

*^++^On good events, a negative score indicates comparative pessimism*.

*Values significantly different from zero (one sample *t* test) are marked with asterisks*.

(*p < 0.05, **p < 0.01, ***p < 0.001).

**Figure 2:**
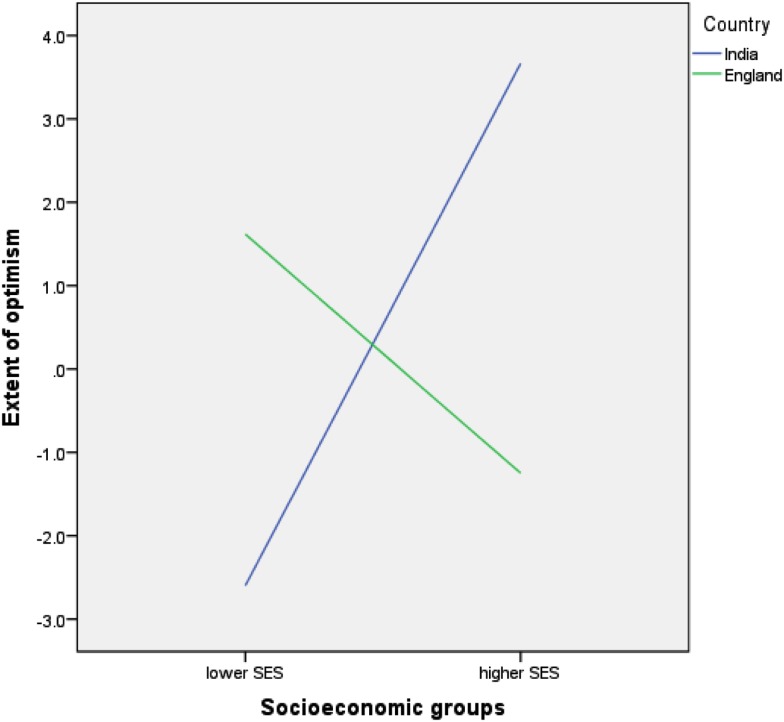
**Relative optimism/pessimism for good events**.

The position regarding comparative optimism for good events was more complex. There were no main effects for nationality [*F*(1,283) = 0.06, *p* = 0.802] or SES [*F*(1,283) = 1.49, *p* = 0.223] but there was an interaction effect between nationality and SES [*F*(1,283) = 1.75, *p* = 0.001]. Higher SES Indians were comparatively optimistic for good events, on average expecting their chances of experiencing these events as 3.7% more than people like them. This number emanates from 60% assessing their chances across events as being on average 9.5% better than others, 14% estimating their chances to be the same as others, and 26% estimating their chances to be 7.7% worse than others. In contrast lower SES Indians were comparatively pessimistic about good events on average expecting their chances of experiencing these events to be 2.6% less than other people like them. This number emanates from 43% assessing their chances across events as being on average 12.3% less than others, 9% estimating their chances to be the same as others, and 48% estimating their chances to be 5.6% better than others. Averaging across events, higher and lower SES English participants showed neither optimism nor pessimism for good events. See Figure 2. Optimism/pessimism scores for good events did not vary by gender or by task order/questionnaire version.

### Optimism/pessimism for individual items

Tables 2 and 3 show responses to the individual items for bad events according to average difference between expectation of event for self and for other, where optimism is indicated by a negative score – i.e., the expectation is that the chance for self is *x* percent less likely than the chance for other. For any given item the average will be comprised of the difference scores of three groups of respondents: those who were optimistic, those who were neither optimistic nor pessimistic (and thus score zero), and those who were pessimistic. For example, in the case of the lower SES Indian sample and the item “committing suicide,” the average difference score of −10.4% emanates from 51% participants stating that their chances were on average 22.5% less than others, 42% stating their chances were the same as others, and 7% stating that their chances were on average 14.3% more than others. Tables 4 and 5 show responses to the individual items for good events according to average difference between expectation of event for self and for other, where optimism is indicated by a positive score – i.e., the expectation is that the chance for self is *x* percent more likely than the chance for other, and pessimism is indicated by a negative score – i.e., the expectation is that the chance for self is *x* percent less likely than the chance for other.

**Table 2:** **Bad events: comparative likelihood estimates, India**.

Bad events	India							
Socioeconomic status	All, *N* = 200		Lower, *n* = 100			Higher, *n* = 100		
	% Chance for other		% Self-other difference^1^		Ratio^2^	% Self-other difference^1^		Ratio^2^
	Mean	SD	Mean	SD		Mean	SD	
Committing suicide	16.58	19.61	−10.42***	16.14	7.00***	−11.10***	17.75	29.00***
Divorce	17.68	18.40	−9.80***	22.34	7.14***	−10.40***	12.14	57.00***
Going bankrupt	20.52	20.61	−3.40	18.01	2.29**	−8.80***	16.10	10.60***
Early death of spouse/partner	23.00	20.64	−3.78*	17.67	1.89*	−4.44**	17.36	3.20***
Son/daughter having a serious illness	26.36	17.78	−5.05***	15.46	2.39***	−3.44**	12.54	3.22***
Serious accident on public transport	27.26	19.68	−2.42	14.08	1.80*	−6.33***	14.24	6.20***
Being burgled	29.64	22.00	−7.63***	20.73	2.82***	−7.55***	17.06	4.63***
Getting cancer	30.20	20.45	−4.80**	17.71	2.05**	−5.35***	13.80	3.80***
Heart attack before 60	33.32	20.00	−5.10**	18.94	1.72*	−5.35**	20.32	3.00***
Financial problems	38.01	24.74	−3.57	26.02	1.33	−9.30***	19.29	4.50***
Being run over	55.13	23.63	−3.88	22.00	1.76*	−5.36***	16.33	4.44***
Average	26.09	12.81	−5.00***	8.23		−6.72***	8.32	

^1^% self-other difference: mean values significantly different from zero (one sample *t* test) are marked with asterisks (*p < 0.05, **p < 0.01, ***p < 0.001).

^2^Ratio of optimists to pessimists: values significantly different from 1 (chance distribution, χ^2^ test) are marked with asterisks (*p < 0.05, **p < 0.01, ***p < 0.001).

**Table 3:** **Bad events: comparative likelihood estimates, England**.

Bad events	England							
Socioeconomic status	All, *N* = 87		Lower, *n* = 47			Higher, *n* = 40		
	% Chance for other		% Self-other difference^1^		Ratio^2^	% Self-other difference^1^		Ratio^2^
	Mean	SD	Mean	SD		Mean	SD	
Committing suicide	18.24	17.67	−6.96*	20.09	4.60***	−2.05	19.24	2.67**
Divorce	43.57	22.95	−12.27**	28.81	3.00**	−14.75**	27.73	4.20**
Going bankrupt	23.95	20.42	−4.78*	15.84	3.00**	−1.00	22.28	3.00*
Early death of spouse/partner	36.27	19.80	0.00	18.18	1.50	0.77	12.48	1.50
Son/daughter having a serious illness	29.51	21.38	−6.05*	17.95	2.80*	−6.84**	14.16	12.00**
Serious accident on public transport	26.16	21.48	−0.87	16.40	1.29	−1.75	8.74	3.50
Being burgled	54.77	24.43	−2.61	16.21	1.67	−2.00	15.88	2.00
Getting cancer	43.84	20.82	3.70	17.98	0.92	3.00	10.18	0.50
Heart attack before 60	33.37	18.64	−3.91	19.50	2.22*	−5.00*	14.85	3.40*
Financial problems	48.37	21.79	−1.30	23.92	1.00	−2.00	22.78	1.78
Being run over	55.11	19.93	0.44	10.21	0.63	−1.75	8.44	2.00
Average	35.82	13.69	−3.23**	7.97		−3.56**	7.16	

^1^% self-other difference: mean values significantly different from zero (one sample *t* test) are marked with asterisks (*p < 0.05, **p < 0.01, ***p < 0.001).

^2^Ratio of optimists to pessimists: values significantly different from 1 (chance distribution, χ^2^ test) are marked with asterisks (*p < 0.05, **p < 0.01, ***p < 0.001).

**Table 4:** **Good events: comparative likelihood estimates, India**.

Good events	India							
Socioeconomic status	All, *N* = 200		Lower, *n* = 100			Higher, *n* = 100		
	% Chance for other		% Self-other difference^1^		Ratio^2^	% Self-other difference^1^		Ratio^2^
	Mean	SD	Mean	SD		Mean	SD	
Unexpectedly inheriting some money	21.36	19.74	−12.65***	19.52	0.11***	−3.90	21.41	0.22***
Winning the lottery	22.74	21.51	−8.67***	21.11	0.43**	−12.22***	20.08	0.09***
Exotic foreign travel	38.32	27.64	−4.95	27.08	0.57*	13.30***	27.04	3.71***
Son/daughter getting a very good job	40.71	25.70	−8.08***	24.07	0.48**	3.06	22.49	1.63
Good health in old age	46.68	21.88	1.30	25.92	0.87	3.74	22.14	1.71*
Moving to a better house	48.95	26.48	2.32	26.58	1.38	2.10	23.54	1.50
Son or daughter does well at school	49.70	26.13	−0.10	23.33	0.83	8.56***	21.08	2.88***
Living a long life	50.81	22.37	1.24	22.03	1.24	0.40	17.46	1.43
Son/daughter being happily married	52.78	23.81	−1.03	20.96	0.78	7.42**	14.65	5.63***
Success for self or spouse at work	55.13	23.63	0.90	20.77	1.28	10.61***	21.12	4.08***
Being on good terms with relatives	56.03	25.08	2.02	28.67	1.54	10.60***	22.10	5.30***
Average	44.23	14.99	−2.60**	11.55		3.66***	10.61	

^1^% self-other difference: mean values significantly different from zero (one sample *t* test) are marked with asterisks (*p < 0.05, **p < 0.01, ***p < 0.001).

^2^Ratio of optimists to pessimists: values significantly different from 1 (chance distribution, χ^2^ test) are marked with asterisks (*p < 0.05, **p < 0.01, ***p < 0.001).

**Table 5:** **Good events: comparative likelihood estimates, England**.

Good events	England							
Socioeconomic status	All, *N* = 87		Lower, *n* = 47			Higher, *n* = 40		
	% Chance for other		% Self-other difference^1^		Ratio^2^	% Self-other difference^1^		Ratio^2^
	Mean	SD	Mean	SD		Mean	SD	
Unexpectedly inheriting some money	31.53	23.12	−12.17***	24.22	0.18***	−10.00*	24.28	0.17***
Winning the lottery	11.55	16.17	−3.56	18.08	0.31*	−2.82	10.11	0.10**
Exotic foreign travel	57.33	25.73	8.04	30.97	1.62	11.75*	32.73	4.20**
Son/daughter getting a very good job	45.49	22.56	2.79	22.20	1.89	−4.10	25.69	0.91
Good health in old age	55.47	16.50	3.91*	12.06	2.57*	−3.75	16.12	0.79
Moving to a better house	50.00	22.49	−1.09	25.39	0.88	−1.00	24.68	1.00
Son or daughter does well at school	50.39	23.38	5.85	25.19	2.25*	−2.97	28.54	1.75
Living a long life	58.59	18.53	3.70	18.81	2.38*	1.03	17.80	1.20
Son/daughter being happily married	43.00	20.65	1.67	17.06	1.67	−4.21	18.21	0.78
Success for self or spouse at work	55.12	19.93	0.87	17.70	1.56	−2.00	14.36	0.77
Being on good terms with relatives	58.14	20.44	10.00**	20.43	6.25***	6.25	21.68	3.29**
Average	47.86	12.55	1.62	9.77		−1.25	10.59	

^1^% self-other difference: mean values significantly different from zero (one sample *t* test) are marked with asterisks (*p < 0.05, **p < 0.01, ***p < 0.001).

^2^Ratio of optimists to pessimists: values significantly different from 1 (chance distribution, χ^2^ test) are marked with asterisks (*p < 0.05, **p < 0.01, ***p < 0.001).

Tables 2– 5 also display responses for each item in terms of the ratio of optimistic to pessimistic responses (optimism indicated by a score above 1.0, and pessimism indicated by a score below 1.0). The ratio scores give more transparent information on score distribution than is evident from the standard deviation of the average difference scores. For example in the case of “Heart attack before 60,” three times as many of higher SES Indian participants expressed relative optimism as compared to relative pessimism, whereas less than two times as many of lower SES Indian respondents did so. Also, by not taking account of the extent of optimism and pessimism, the ratio scores are not subject to the averaging problem where, as in the case of the lower SES Indian group’s good event scores, an average difference score of −2.6% came about through a slightly smaller group of respondents being very pessimistic outweighing a slightly larger group of participants being mildly optimistic. (In the case of the higher SES Indian participant response for good events, and all four groups’ responses for bad events, a significant deviation from zero arises by there being more optimists than pessimists, showing approximately equal degrees of optimism or pessimism respectively).

### Event desirability and event controllability

As described in the section “Materials and Methods,” four further groups of participants (matched for nationality, location and SES) judged the events for desirability and controllability. The desirability rankings of the 11 bad event items was very similar between nationalities [*rho*(9) = 0.89, *p* < 0.001] and did not vary by SES. Early death of spouse and suicide were rated as the very worst events and financial difficulties and being burgled as the least bad events. Within nationalities the ranking of the 11 good event items did not vary by SES, but the desirability rankings of the good events between nationalities was not similar [*rho*(9) = 0.48, *p* = 0.482]. For Indian participants the highest ranking events were “success for self or spouse at work” and “son or daughter doing really well at school” and the lowest ranking events were “winning the lottery” and “unexpectedly inheriting some money.” For English participants the highest ranking events were “good health in old age” and “son or daughter being happily married” and the lowest ranking events were “moving to a better house” and “exotic foreign travel.”

In terms of absolute amount of controllability (where 1 signified “impossible to control” and 4 signified “certainly possible to make this happen/prevent this happening”), good events were rated 2.44 (*SD* = 0.35, *N* = 84) and this did not vary by nationality or by SES as simple main effects or in interaction. On average, bad events were rated 2.38 (*SD* = 0.40, *N* = 88) but in this case English participants rated such events as slightly less controllable (*M* = 2.21, *SD* = 0.37) than did Indian participants [*M* = 2.48, *SD* = 0.38; *F*(1,85) = 10.65, *p* = 0.002]. These judgments were not affected by SES as a main effect or in interaction with nationality.

The ranking of bad event items for controllability was very similar between nationalities (*rho*(9) = 0.77, *p* = 0.005), and did not vary by SES. The highest controllability scores were given to committing suicide, going bankrupt and divorce; low controllability scores were given to son/daughter’s serious illness, early death of spouse, and cancer. Within nationalities, the ranking of good event items for controllability did not vary by SES, but the rankings between nationalities were not similar [*rho*(9) = 0.39, *p* = 0.233]. Indian participants gave relatively high controllability scores to “son/daughter doing well at school” and “son/daughter being happily married,” and a relatively low controllability score to “exotic foreign travel.” In contrast English participants gave relatively high controllability scores to “exotic foreign travel” and “moving to a better house,” and a relatively low controllability score to “son/daughter being happily married.”

A further analysis of the perception of good events was conducted in light of the apparent nationality differences in how the items were ranked for desirability and for controllability. As described above, the Indian informants who evaluated the suitability of Weinstein’s (1980) list of good and bad events, nominated 10 new items, five of which consisted of events in the lives of close relatives rather than directly in the life of self. Examples of such events were “son/daughter serious illness” and “son/daughter getting a good job.” As a consequence of these additions, the list of 11 good event items was composed of five items in the lives of close relatives and six items more explicitly in the life of self. A repeated measures ANOVA indicates that while both Indian and English participants ranked the good event items involving others as more desirable than the items involving self [*F*(1,90) = 32.21, *p* < 0.001], there is a suggestion that this difference was more striking for Indian than for English participants [average difference in rank between items involving others and items involving self: India = 2.08, *SD* = 2.23; England = 1.07, *SD* = 2.74; *F*(1,90) = 3.65, *p* = 0.059]. A repeated measures ANOVA indicates that while both Indian and English participants rated the good event items involving others as more controllable than the items involving self [*F*(1,90) = 69.36, *p* < 0.001], this difference was more pronounced for Indian than for English participants [average difference in controllability scores between items involving others and items involving self: India = 0.70, *SD* = 0.58; England = 0.40, *SD* = 0.59; *F*(1,90) = 5.16, *p* = 0.026]. SES was not a significant factor in either of the two repeated ANOVAS.

### Relationship between event frequency, desirability, controllability, and optimism

Tables 2– 5 show the average estimates for bad and good event frequency given by participants when considering the chances of the “other.” Bad events were considered to be less frequent than were good events, and this was true for Indian and English participants. Across bad events, Indians gave on average lower frequencies (*M* = 26.1%, *SD* = 12.81) than did English participants [*M* = 35.8%, *SD* = 13.69; *F*(1,283) = 28.25, *p* < 0.001]. This did not vary by SES, nor was there an interaction between SES and nationality. Across good events, Indians on average estimated slightly lower frequencies (*M* = 44.3%, *SD* = 14.99) than did English participants [*M* = 47.9%, *SD* = 12.55; *F*(1,283) = 3.93, *p* = 0.049]. This did not vary by SES, but the interaction between SES and nationality approached significance [*F*(1,283) = 3.45, *p* = 0.064] suggesting that the lower Indian frequencies were a feature of the lower SES Indians’ judgments (40.7%, *SD* = 16.48) and not of the higher SES Indians’ judgments whose frequency scores averaged at 47.3% (*SD* = 12.89) and were thus very similar to the judged frequencies offered by the English participants.

Factor analysis was used to explore the relationship between event frequency (for “similar others”), event controllability and event desirability as initial multiple regressions looking at the relationship of these three variables to optimism/pessimism for bad and good events separately, revealed high levels of multicollinearity between the three independent variables (indicated by pair-wise correlations above 0.80 and VIF values >5). A Principal Components Factor Analysis using varimax rotation of the judgments of bad events yielded two factors with Eigen values greater than 1.0 which accounted for 88% in variance in the data. Factor 1 explained 47% of the variance and comprised controllability and desirability. Factor 2 explained 41% of the variance and comprised event frequency. A series of four multiple regressions, as displayed in Table 6, showed that these factors relate to optimism difference scores but only in the case of higher SES Indians. For this group higher optimism was expressed about events which were judged as more controllable and less undesirable (i.e., ranked as less bad among bad events). For example, participants showed high levels of optimism about divorce (i.e., thought that their chances of experiencing this event were less than the chance for similar others). As an event divorce was rated as relatively controllable and not as undesirable as events such as getting cancer. While still optimistic, higher SES Indians were less optimistic about items such early death of spouse, regarded as relatively uncontrollable and highly undesirable. For higher SES Indians event frequency, unrelated to desirability or controllability, negatively predicted relative optimism although less powerfully than desirability/controllability. As an example, higher levels of optimism were expressed in relation to going bankrupt (judged as a low frequency event for “similar others”) and lower relative optimism was expressed in relation to having a heart attack before 60 (judged as a relatively higher frequency event for “similar others”).

**Table 6:** **Regression coefficients showing the relationship of event controllability, desirability, and frequency to comparative optimism/pessimism**.

Country	India				England			
Socioeconomic status	Lower		Higher		Lower		Higher	
	*t*	*p*	*t*	*p*	*t*	*p*	*t*	*p*
**BAD EVENTS**								
Factor 1, Controllability/desirability	1.30	0.231	3.41	0.009	1.21	0.261	−0.59	0.574
Factor 2, Frequency	−1.04	0.328	−2.31	0.050	−0.56	0.592	−0.15	0.887
Regression *F*(2,10)	1.96	0.203	8.73	0.010	0.81	0.480	0.17	0.850
**GOOD EVENTS**								
Factor 1, Controllability/desirability/frequency	4.66	0.001	3.49	0.007	3.31	0.009	1.69	0.130
Regression *F*(1,10)	21.68	0.001	12.19	0.007	10.95	0.009	2.85	0.130

A Principal Components Factor Analysis of the judgments of good events yielded only one factor with an Eigen value greater than 1.0 which accounted for 70% of the variance in the data. All three variables loaded highly on the factor, indicating that good events judged to be more frequent for “similar others” were also judged to be more desirable and more controllable. As can be seen in Table 6, multiple regressions indicated that this factor was significantly related to optimism/pessimism in three of the four groups. For example, higher SES Indians expected to be less likely than similar others to win the lottery (a less valued event judged as relatively uncommon and uncontrollable), but they expected to be more likely than similar others to experience success at work (a highly valued event judged to be relatively common and controllable). The relationship between the controllability/desirability/frequency factor and optimism/pessimism was particularly strong for the lower SES Indian group who, as the final row of Table 4 makes clear, on average were comparatively pessimistic about their chances of experiencing good events. In their case a positive correlation between the factor and optimism/pessimism indicates that they were particularly pessimistic regarding the rarer, less controllable, and comparatively less valued events (such as “unexpectedly inheriting some money”) and neither optimistic or pessimistic about events regarded as the more frequent, more controllable, and more valuable (such as “success for self/spouse at work”).

Taken as a whole the results show that, for three of the four groups studied, neither event controllability/desirability nor frequency played a part in the determination of relative optimism for bad events. For good events however, gradations in relative optimism/pessimism related to item controllability/desirability/frequency for three of the four groups studied. More highly valued events were judged more controllable and more frequent than less valued events, and were more subject to relative optimism (or as in the case of the lower SES Indian sample were less subject to relative pessimism).

## Discussion of Results

The primary aim of the study was to investigate unrealistic optimism in two different cultures, with participants drawn from two different socioeconomic groups within those cultures. For bad events, participants from both socioeconomic groups in India and England showed comparative optimism as they considered their chances of experiencing such events to be less than the chances of other people like themselves experiencing those events. This was so whether optimism was judged by the average of respondents’ self-other difference scores per event, or the average of those scores across 11 events, or in terms of the ratio of optimists to pessimists per event. Further, Indian participants showed greater optimism regarding bad events than did English participants. The picture changed however for good events. Although not optimistic on every item, the higher SES Indian group on average continued to show comparative optimism expecting to be more likely to experience these events than other people like themselves. But in contrast, the lower SES Indian group on average showed pessimism regarding their chances of experiencing good events which came about through being outright pessimistic on some items and neither optimistic or pessimistic on the remaining items. Across good items, English participants of both socioeconomic groups averaged to be neither optimistic nor pessimistic.

Much of the research on comparative optimism has investigated respondents’ expectations about their likelihood of experiencing negative life events. It is this kind of research, particularly when respondents are invited to make separate judgments about their own and “the other’s” chances, that finds low or zero rates of comparative optimism among those from East Asian cultures (Heine and Hamamura, 2007; Rose et al., 2008). The current study, despite asking participants to make separate judgments about their own chances and the chances for others, found that Indian participants showed high rates of comparative optimism for negative events. Matsumoto (2007) has lamented the tendency of researchers taking “findings from one or a few countries from the Asian continent, especially East Asia, and making generalized interpretations about ‘Asia’ and ‘Asians’ in general” (p. 47). The clear finding in the current study that Indian participants showed unrealistic optimism on negative items, and that wealthier Indian participants also showed unrealistic optimism on positive items, demonstrates that the lack of unrealistic optimism found in some East Asian cultures is not generalizable to other Asian cultures.

The most frequent explanation of cultural differences in unrealistic optimism relates optimism to the tendency to self-enhance thought to be characteristic of individualistic cultures comprised of independent selves. Lack of unrealistic optimism is held to relate to a reluctance to self-enhance thought to be characteristic of a collectivist culture comprised of interdependent selves (Heine, 2003). The high rate of unrealistic optimism shown by the Indian participants in the current study makes it clear that it is possible to demonstrate self-enhancement and yet be from a collectivist Asian culture. One response to this finding might be that perhaps India is not a very collectivist culture. Indeed Sinha and Tripathi (1994) note that even though India does not occupy a very low place on the Individualism-Collectivism scale, the categorization of India as collectivist has “somehow gotten stuck to the Indian culture” (p. 124). Citing Tripathi (1988), Mishra (1994) proposes that for Indians in-group orientation may not signify collectivism in the “normal” sense but be used as a strategy for enhancement of the self or one’s own family.

A different interpretation of the results of the current study is that collectivism in India takes a different form from collectivism in East Asian cultures and is not associated with self-effacement. On the basis of a cultural analysis and questionnaire survey responses Sinha and Tripathi (1994) propose that the Indian form of collectivism contain important strands of individualism, and that whether collectivist or individualist values determine behavior among Indians is very context dependent. In a similar vein, in a discussion of the “Indian mindset” and negotiating behavior, Kumar (2004; citing Sinha and Kanungo, 1997) suggests that while managers’ “primary mode of behavior reflects the prevalence of traditional Hindu values such as collectivism and high power distance, the secondary mode reflects the inculcation of values such as individualism and pragmatism” (p. 42). Also relevant to the current study’s demonstration of unrealistic optimism among Indian participants is Kumar’s (2004) depiction of a mode of thought he labels as “Brahmanical idealism,” characterized by over-optimistic confidence and “wishful thinking that may be divorced from empirical reality to an excessively high degree” (p. 45). Rather than classify cultures as collectivist or individualistic, and selves as interdependent or individualist, it may be more useful to employ Kagitcibasi’s (2005) twofold orthogonal dimensions of agency (autonomy-heteronomy) and interpersonal distance (separation-relatedness), and describe the Indian self as autonomous-related in comparison to the Western autonomous-separate self.

As already discussed, Indian participants showed greater optimism than did English participants on negative events, and on positive events higher SES Indians also showed optimism and were the only group to do so. Broadly this indicates a greater degree of optimism and self-enhancement among Indian than English participants. Loughnan et al. (2011) proposed that self-enhancement relates to income inequality and their measure of choice was the Gini coefficient. However in explaining the apparent connection between self-enhancement and income inequality, Loughnan et al. (2011) suggest that “it is unlikely that economic inequality *directly* leads to biased self-perception” (p. 1257; italics our own). Rather they argue that self-enhancement relates to intervening factors that result from socioeconomic differences. Loughnan et al. (2011) develop their explanatory model with reference to Wilkinson and Pickett’s (2010) proposal that living in an unequal society engenders social evaluation anxiety which itself increases the tendency to self-promote and self-enhance. If there are such psychological consequences of living in an unequal society, these are likely to be driven by perceptions of inequality rather than inequality as measured by statisticians. A society will “feel” very unequal when, for example as in India, a substantial proportion of the population live below the international poverty line alongside other members of the same society living in extreme affluence. It is also the case that although social stratification is a historic and current feature of English society, India’s social structure is characterized not only by socioeconomic class but also by elaborate hierarchy and differentiation in the form of caste (Srinivas, 1996; Desai and Dubey, 2011). India also scores much higher than the UK on Hofstede’s Power Distance dimension thought to measure the extent to which hierarchy and inequality is accepted in a society^6^. Further, Bardhan (2009) has suggested that a like-with-like measure of income (as opposed to consumption) inequality would give India a Gini coefficient of income inequality of 53.5%, i.e., much higher than the UK’s score of 36%. In many senses, inequality and social differentiation in India is arguably greater than in the UK, and the higher rate of unrealistic optimism shown by Indian as compared to English participants thus supports Loughnan et al.’s (2011) general proposition that self-enhancement relates to inequality in society^7^.

An interesting aspect of the data is the contrasting profiles of the higher and lower SES Indian groups. The higher SES Indians were the only group in the study to show optimism for both bad and good events. For this group both bad and good events deemed to be more controllable attracted greater optimism. This supports McKenna’s (1993) proposition view that unrealistic optimism is not so much a general generalized expectancy for a positive outcome but more closely related to the extent to which participants consider that they have control over the outcome. But it also suggests that the illusion of control is as an influential factor in the mindset of Indians as it has been held to be in the Western mindset, and accords with Shweder’s (2008) observation that, contrary to what is often believed by Westerners, Hindus do have a strong sense of agency^8^^,^^9^.

While optimistic bias may promote general psychological well-being (Taylor and Brown, 1988), there is evidence that it may at the same time deter those at risk from engaging in health promoting behaviors (Dillard et al., 2006). In the current study, among the higher SES Indians who rated their chances of having a heart attack as being different from that of others, 75% displayed optimism, with the modal response being that their own chance was 20% less than that others. Since for this group optimism regarding having a heart attack correlated very highly with optimism on many other items such as not going bankrupt, and not having an accident while on public transport, it can be safely interpreted that the optimism relates to a general psychological orientation rather than reflecting the practice of actual health promoting behaviors. Reddy and Yusuf (1998) suggest that community awareness of the dangers of cardiovascular disease is not high in countries such as India, and further that “the transition toward becoming industrial market economies is unleashing consumer aspirations that impatiently seek an affluent and indulgent lifestyle” (p. 601). The authors speculate that “messages of moderation may not be welcome during such periods of change” (p. 601). The current study suggests that in India as in the UK, unrealistic optimism is likely to play a part in people’s inattention to or even rejection of health messages.

A striking feature of the current study is that although lower SES Indian participants showed unrealistic optimism on bad events, they did not show it on good events. In fact, their average score on good events was pessimistic which was in stark contrast to higher SES Indian participants who averaged as optimistic for the same events. For lower SES Indians (as was the case for two of the three other groups, and for the fourth group the trend was in the same direction) optimism for the good events was related to events being judged as more controllable, more highly valued and estimated as more frequent. But as has already been pointed out for lower SES Indians, “optimism” on good events was not optimism, but merely lack of pessimism. Both Indian groups regarded events such as work success, moving to a better house, son/daughter being happily married as frequent, valued, and relatively controllable^10^. But while higher SES Indians regarded their chances of experiencing such events as being greater than that of similar others, lower SES Indians did not. A fear of the evil eye is one possible explanation for lack of self-enhancement on good items. Anthropologists and psychologists have described the evil eye belief, common in India, as the fear of one’s good fortune arousing jealousy in others and consequently bringing bad luck to the self (Pocock, 1973; Anandalakshmi, 1978; Shweder, 2008). The widespread sale of amulets and the frequent performance of rituals to avert the evil eye testify to the strength of the belief in the evil eye in modern India. Such a belief would tend to encourage self-effacement in estimating one’s chances of experiencing the good things in life, particularly regarding events involving one’s children^11^. Further research is needed to examine whether the prevalence of such beliefs varies between different socioeconomic communities in India, and more generally whether the self-effacement in regard to good events shown by the poorer Indians in this study is merely a “performance” – i.e., is driven by a wish not to publically claim the prospect of good fortune, in a manner reminiscent of modesty, which Kurman (2003) suggests is a plausible determinant of self-effacing behavior in some collectivist cultures.

An alternative explanation for lack of apparent optimism on good items is that for some people lack of optimism – or indeed comparative pessimism – particularly in regard to common valued events, reflects a depressive state of mind^12^. Although employed and housed, the lower SES group live much closer to the economic margin and destitution than do the higher SES Indian group. Given the well established relationship between material hardship, financial strain and depression (Dohrenwend et al., 1992; Lorant et al., 2007), that depression might be more common among the lower SES group in India is unsurprising. A large scale epidemiological study in Chennai attests to the prevalence of depression in urban India and its relation to economic hardship. Self-reported depression (measured by a locally modified version of the Patient Health Questionnaire) was as high as 19.3% in the low income group as compared to 5.9% in the higher income group (Poongothai et al., 2009). The authors of the Chennai study conclude that there is a clear need to increase mental health services in India. In the current study nearly half of the lower SES Indian group expressed pessimism on as many as five or more of the 11 positive items. That this group may be especially vulnerable to depression tallies with ethnographic depictions of urban “locally oriented middle class” Indians who, notwithstanding rapid economic growth in India during the last three decades, continue to experience limited economic opportunities for themselves and their children (Derné, 2008), and feel insecure in the face of the threat of “real impoverishment” (Shurmer-Smith, 2000, p. 52).

### Methodological considerations and limitations of the current study

Chambers and Windschitl (2004) and Rose et al. (2008) argue that a powerful cognitive bias operates in judgment tasks, in that people pay relatively careful attention to event base rate information when judging their own probability of experiencing an event, but overestimate the base rate for rare events and underestimate it for common events when judging the probability of others experiencing the same event. The operation of this egocentric bias is held to lead to unrealistic optimism for infrequent negative events and unrealistic pessimism for frequent negative events. In the current study the frequency of bad event items did affect optimism, although the effect was relative rather than absolute and only evident in one group. For higher SES Indians greater optimism was shown for the least frequent bad events. They did not show pessimism for the more frequent bad events, although they did show less optimism for such events. Further research is needed to examine the relationship between optimism and event frequency, by varying events by their absolute frequency as well as by their frequency relative to each other. In the current study even the rarer bad events were judged to be quite common. This contrasts with studies which have used events such as being struck by lightning.

There was stronger evidence of base rate bias in optimism/pessimism for good events. In the case of good events a base rate bias of the kind suggested by Chambers and Windschitl (2004) and Rose et al. (2008) would produce pessimism for rare events and optimism for frequent events. This pattern was found for three of the four groups in the current study. However the data cannot be taken as giving clear support for the base rate explanation of unrealistic optimism as event frequency for good events was confounded with rank and controllability. The number of events was insufficient to permit the disentanglement of frequency, desirability and controllability in the manner of Chambers et al. (2003).

Harris and Hahn (2011) have recently and controversially suggested that the phenomenon of unrealistic optimism is neither a genuine cognitive nor motivational risk bias but is only an artifact of the kinds of response scales conventionally used in unrealistic optimism studies. They are particularly concerned with the risk perception of rare negative events, and suggest that the few respondents who for some reason have a rational reason to be pessimistic regarding the risk in question (for example through a known genetic predisposition to a rare disease) do not get a chance to register the full extent of their pessimism as the design of response scales does not enable their responses to balance the more moderate optimism of the majority who have a reason to be optimistic (for example by knowing they are not in an at risk group for the disease in question). It is unlikely that the current study is subject to this problem as few of the events were very rare or of the kind associated with firm probability forecasts.

In this, as in many studies in social psychology, participants are requested to respond to a set of stimuli thought to represent categories of theoretical interest, and much may depend on the stimuli chosen by the researchers (Judd et al., 2012). In the current study Weinstein’s original list of good and bad events was developed by a small group of Marathi-English speaking Indian advisors to ensure that the list was suitable for use by Indian participants. It was notable that many of the substitutions consisted of events in the lives of close relatives rather than in the life of self, and that this disproportionately affected the list of good rather than bad events. While Indian and English participants ranked the good event items involving others as more desirable and more controllable than the good items involving self, this tendency was more striking for Indian than for English participants. It is possible that the English participants’ lack of optimism for good events relates in part to the list being somewhat culturally alien.

Noting that many studies only ask participants to make judgments of life events chosen by the researcher, Hoorens et al. (2008) invited participants to nominate their own items. Their data demonstrated comparative optimism in that student participants listed more desirable and fewer neutral and undesirable events in their future in comparison to the average student’s future, and also showed comparative optimism in their estimates of the likelihood of those events. Many of the events listed were similar to standard researcher generated lists, and also showed overlap with some of the new items suggested by Indian participants in the current study (e.g., being involved in a serious accident; early death of spouse/partner). Nevertheless it was notable that three of the 10 new items suggested in India involved money, which brought the number of explicitly financial items to a total of six items, four of which were located in the good event list. All four groups of participants showed pessimism on two good events directly related to finance (“unexpectedly inheriting some money” and “winning the lottery”). Lower SES Indian participants were also pessimistic regarding another financial based event (“exotic foreign travel”) and the only bad item they were not optimistic about was their relative chance of experiencing “financial problems.” But it is unlikely that these financially related events can be held responsible for the lack of optimism shown for good events. Subjecting the four financial good items to Cronbach reliability testing only generated an alpha of 0.374 for the lower SES Indians (and was no higher for any of the other groups) indicating that for individuals pessimism/optimism judgments on financial items do not cohere^13^

In terms of absolute levels of optimism/pessimism on good events, lower SES Indians were also pessimistic about “son/daughter getting a very good job,” and not optimistic on any of the remaining good event items even though some of those items had been judged by a comparable group of participants as controllable – i.e., as reasonably or even certainly possible to make happen. Further research is needed to explore what is meant by “controllable,” as events may be being viewed as controllable by others but not by the self. In this case the lower SES Indian participants’ striking lack of optimism about good events in comparison to the higher SES Indian participants may indeed signal a somewhat dejected approach to the future. As already indicated, averaging across all good items, 43% of the lower SES Indian group were pessimistic about their relative chances of experiencing those items in comparison to people like themselves. The comparable figure among the higher SES Indian group was only 26%.

It was considered important to sample from different socioeconomic groups not only in relation to the study’s particular interest in inequality but also because psychology in general has been guilty of oversampling from a very restricted age and educational pool with possible consequent problems for the generalizability of studies’ results (Henrich et al., 2010). The use of different items and different rating scales renders cross-study comparisons problematic. However the English middle aged participants in the current study showed a clear pattern of unrealistic optimism on negative events, very similar to patterns of unrealistic optimism found in adult and student samples in North America. The English participants were randomly selected from non-academic employees in lower managerial and intermediate occupations at a university in the south of England. With the aim of matching occupation and status to the English groups, Indian participants were recruited from housing associations in two suburban areas, one middle class and one lower middle class, in Maharashtra. There is no reason to regard any of the four groups as atypical, but only further research can establish the generality of the findings of the current study to other parts of England and India, and to non-Hindus in India. More interesting still would be to study unrealistic optimism among participants more economically deprived than those sampled in the current study.

## Conclusion

A number of universalist discourses surround research on unrealistic optimism. Neuropsychologists such Sharot et al. (2011) regard the optimism bias as a “pervasive human trait” motivating adaptive behavior in the present toward a future goal. For such authors the model is of “the tendency (of the healthy brain) to generate images of positive future events” (Sharot et al., 2007, p. 102), and a lack of optimism is thought to indicate poor mental health. A universalist approach is also taken by those who emphasize the role of cognitive biases in social comparative judgments (Windschitl et al., 2008).

Motivational explanations for unrealistic optimism have been more common among researchers whose prime interest is in cultural comparison. A key focus has been the relationship of self-enhancement and self-effacement to selves contrasted in terms independence/interdependence and societies contrasting in individualism/collectivism. In the years following the work of Hoftsede (1980, 2001) and Triandis (1988, 1995), it has been suggested that the collectivism of some cultures such as Japan has been overstated and, in any event, is locally variable and has been subject to change (Matsumoto, 1999, 2002; Takano and Sogon, 2008; Yamawaki, 2012). Such a point of view might be interpreted as rendering collectivism/individualism (and the interdependent/individual self construct) less useful as explanations for east/west differences in unrealistic optimism than formerly thought. Nevertheless, there are “profound cultural differences in the ways people come to understand themselves” (Heine, 2012, p. 195), and categorizations such as interdependence/independence and collectivism/individualism have been and will continue to stimulate the formation of hypotheses about culture and behavior.

The current research used a methodology which to date has found less or no unrealistic optimism in certain East Asian collectivist cultures. But in this study Indian participants showed even higher levels of unrealistic optimism, particularly for bad events, than did English participants. Broadening the field of enquiry to other Asian societies, such as India, underlines the complexity of the relationship of self-enhancement and self-effacement to comparative optimism.

## Conflict of Interest Statement

The authors declare that the research was conducted in the absence of any commercial or financial relationships that could be construed as a potential conflict of interest.

## Footnotes

^1^Whether the peoples of India can be described as sharing a culture has been the subject of intense debate (Ramanujan, 1989; Khilnani, 1997). On the one hand the geopolitical entity that is India encompasses a variety of religions, languages, and cultural practices. On the other hand in their observations of India, authors ranging from psychoanalysts such as Roland (1988) to anthropologists such as Alvi (2007) find a cultural commonality beneath the layered and apparently divergent characteristics. It is noteworthy that Jawaharlal Nehru, the architect of modern India, while commenting on the tremendous diversity of India made much of its “essential unity” and “common outlook on life” (Nehru, 1961, p. 59).

^2^On Hofstede’s I/C scale India scores 48 which is very similar to Japan’s score of 46, but higher than the scores of Hong Kong 25, China 20, South Korea 18, and Taiwan 17. The UK’s score is 89 (http://geert-hofstede.com/dimensions.html).

^3^On the specific topic of hierarchy, Srinivas (1962) noted that the phenomenon of caste “cuts across religious divisions – it is not only Hindus who are segmented into castes but also Jains, Sikhs, Muslims, and Christians (p. 103). Indeed Srinivas (1962) suggested that “The institution of caste provides a common cultural idiom to Indians: wherever one may be in India, one is in a universe of caste” (p. 103).

^4^The Gini income coefficient is a measure of the deviation from a perfectly equal distribution of income among individuals or households within a country, where 0 represents absolute equality and 100 represents absolute inequality. On this measure India’s coefficient of 36.8% is very similar to the UK’s coefficient of 36.0% (United Nations Development Programme, 2010).

^5^The major five self-identification ethnic categories used in the UK Census are White; Mixed/Multiple ethnic groups; Asian/Asian British (Indian, Pakistani, Bangladeshi, Chinese, Other Asian); Black/African/Caribbean/Black British; and Other ethnic group (Office for National Statistics, 2011).

^6^Power distance scores: India, 77; UK, 35 (http://geert-hofstede.com/dimensions.html).

^7^In Loughnan et al.’s (2011) own study power distance did not add to the variance in self-enhancement explained by the Gini coefficient, but the authors do not report whether power distance predicted self-enhancement in a pair-wise comparison.

^8^In Shweder’s view “the “Western” notion that Hindus in the “East” are passive, lacking in a sense of agency or internal control and resigned to their fate, is one of the greatest of all cross-cultural misunderstandings” (2008, p. 76).

^9^See Savani et al. (2008) for a discussion of the different forms that agency may take in Indian and North American cultural contexts.

^10^That Indian participants regarded “a son/daughter being happily married” as “controllable” reflects the arranged marriage system common in India. In a large scale survey involving 2496 women in Maharashtra aged 25–49, respondents indicate 3% of marriages had been self-arranged; 34% jointly arranged by parent and self; 34% arranged by parents with self having some say/consent in the choice; and 29% parent-arranged marriages where self had no say in choice of partner (Banerji et al., 2008). In arranging a marriage, by choosing a suitable spouse parents hope to ensure the well-being of their child (Medora, 2003).

^11^Parents are particularly anxious not to attract the evil eye by over-praising a child’s beauty or achievement (Roland, 1988).

^12^In an exploration of cognitive biases in non-human animals, Matheson et al. (2007) studied “optimistic” response biases in wild-caught captive European starlings, trained to discriminate between two signals differentially associated with a better or less good outcome (i.e., instant or delayed food). Birds whose cages were bigger and enriched showed greater optimism in the face of an ambiguous signal in comparison to birds in smaller and unenriched cages. The authors liken the response of the later group to that of “depressive realism” in humans.

^13^The Cronbach’s alpha for all six financial items (two bad, four good) was also low: 0.306 whole sample, 0.208 lower SES Indian group.
